# Human Mature Adipocytes Express Albumin and This Expression Is Not Regulated by Inflammation

**DOI:** 10.1155/2012/236796

**Published:** 2012-05-15

**Authors:** Maria Luisa Sirico, Bruna Guida, Alfredo Procino, Andrea Pota, Maurizio Sodo, Giuseppe Grandaliano, Simona Simone, Giovanni Pertosa, Eleonora Riccio, Bruno Memoli

**Affiliations:** ^1^Department of Nephrology, University Federico II of Naples, 80131 Naples, Italy; ^2^Nutrition Section, Department of Neuroscience, University Federico II of Naples, 80131 Naples, Italy; ^3^Department of General Surgery, University Federico II of Naples, 80131 Naples, Italy; ^4^Department of Nephrology, University of Bari, 70124 Bari, Italy

## Abstract

*Aims*. Our group investigated albumin gene expression in human adipocytes, its regulation by inflammation and the possible contribution of adipose tissue to albumin circulating levels. *Methods*. Both inflamed and healthy subjects provided adipose tissue samples. RT-PCR, Real-Time PCR, and Western Blot analysis on homogenates of adipocytes and pre-adipocytes were performed. In sixty-three healthy subjects and fifty-four micro-inflamed end stage renal disease (ESRD) patients circulating levels of albumin were measured by nephelometry; all subjects were also evaluated for body composition, calculated from bioelectrical measurements and an thropometric data. *Results*. A clear gene expression of albumin was showed in pre-adipocytes and, for the first time, in mature adipocytes. Albumin gene expression resulted significantly higher in pre-adipocytes than in adipocytes. No significant difference in albumin gene expression was showed between healthy controls and inflamed patients. A significant negative correlation was observed between albumin levels and fat mass in both healthy subjects and inflamed ESRD patients. *Conclusions*. In the present study we found first time evidence that human adipocytes express albumin. Our results also showed that systemic inflammation does not modulate albumin gene expression. The negative correlation between albumin and fat mass seems to exclude a significant contributing role of adipocyte in plasma albumin.

## 1. Introduction

Adipose tissue, once just believed a passive organ storing excess energy, is now considered a real endocrine organ, with an important role in the regulation of homeostatic systems [1]. It secretes various proteins that can be classified into molecules acting on metabolic processes like glucose homeostasis and insulin sensitivity, and into inflammatory molecules like interleukin 6 (IL-6), interleukin 1*β*, Tumor Necrosis Factor-*α*, C-reactive protein (C-RP), Leptin, and Adiponectin [2]. The adipose tissue may account for 20–25% of systemic IL-6 circulating levels [3] and seems to be involved in the systemic inflammatory response associated with obesity, insulin resistance, and metabolic syndrome [4].

On this basis, adipose tissue represents a new and fascinating multidisciplinary object of research. We previously found a clear expression of IL-6 receptors and C-reactive protein in human adipocytes obtained from adipose tissue fragments of different districts (subcutaneous and omental of noninflamed and inflamed patients), showing that the adipose tissue, and in particular the adipocyte, was not only responsible for generating an inflammatory state per se but it also represents a target, by itself, of systemic inflammation [5]. We observed, in fact, that the adipocyte, stimulated by IL-6 through its cell receptor produces C-RP, as it happens in the hepatocyte.

Adipose tissue is a mesodermallyderived organ that contains, in addition to adipocytes, a stromal population composed by different cell types, such as pre-adipocytes, stem cells, fibroblasts, and macrophages. Mature adipocytes account for only 40–60% of the whole cell population whatever the type of adipose tissue [6]. Pre-adipocytes are present throughout adult life in adipose tissue and can proliferate and differentiate into mature adipocytes, according to the energy balance; they also share some features with macrophages, such as the capacity of phagocytosis in response to different stimuli [7]. Another important cell population of the adipose tissue is represented by mesenchymal stem cells (MSCs) that are adherent, fibroblast-like, pluripotent, and nonhematopoietic progenitor cells. These cells retain the capacity to undergo into many mesenchymal cell types, including bone cells [8, 9], neuronal cells [10], adipose [11], and muscular [12] tissue *in vitro*, and of note, into hepatocyte-like cells [13–15]. Thereby, both the pre-adipocyte, with a very restricted potential, and the multipotent mesenchymal cell represent adipocyte precursors.

 Albumin, normally produced by the hepatocyte, is the most abundantly circulating protein in blood, where it plays an important role as binding protein and is involved in the regulation of colloid-osmotic homeostasis [16]. Circulating half-life of plasma albumin is 19 to 21 days. It is also a negative acute phase protein and hypoalbuminemia is the result of the combined effects of inflammation, through inhibitory cytokine (especially IL-6), and inadequate protein and caloric intake in patients with chronic inflammatory disease, such as chronic renal failure [17] where it represents a cardiovascular risk factor [18].

Considered the well-known active role of adipocyte in C-RP production and the presumable link between adipocyte and hepatocyte, we aimed this study at investigating if mature adipocytes express the gene of albumin and at evaluating whether systemic inflammation might regulate this expression, as we previously found for C-RP [5]. Furthermore, we speculated whether the adipocyte might contribute to the total circulating levels of albumin, as it happens for IL-6.

## 2. Methods

### 2.1. Quantization of Albumin mRNA and Protein Presence in Adipocytes and Preadipocytes

#### 2.1.1. Patients and Healthy Controls Selection and Adipose Tissue Samples

Human subcutaneous adipose tissue samples were obtained from twelve subjects divided in two subgroups on the basis of a preliminary evaluation of C-RP circulating levels. According to Pepys and Hirschfield [19] we assumed a cut-off level of 3 mg/L, in order to discriminate between inflamed and noninflamed patients. On the basis of C-RP values, we obtained a group of six healthy (noninflamed) subjects, without any clinical symptoms or sign of inflammation, who underwent minor surgery procedures and a group of six patients with chronic inflammatory disease who underwent elective surgical procedures: (a) four for cancer and (b) two patients operated for cholecystectomy.

All enrolled subjects provided written, informed consent, and the Ethics Committee of our hospital approved the study.

To avoid a possible confounding inflammatory effect exerted by obesity or overweight status, inflamed patients and noninflamed controls were BMI matched in both groups of subjects. The presence of other systemic diseases (vasculitis, rheumatoid arthritis, osteoarthritis, and bowel or lung inflammatory disease) was excluded in all subjects (excepted malignancy); in particular, before the definitive inclusion, the possible existence of any immunological disease, malignancy (in the healthy group), and infectious disease was carefully investigated and excluded. No patient was diabetic and none assumed steroid or immunosuppressive therapy.

Blood samples were collected, before surgical procedures, from all studied subjects to obtain serum aliquots. Sera were stored at −80°C until C-RP and IL-6 assays were performed. Adipose tissue biopsies were obtained during the surgical procedures in all 12 subjects and for each patient was obtained a sample of subcutaneous total white adipose tissue and a sample of omental adipose tissue. After removal, to get rid of tissue debris the bioptic material was washed twice with warm sterile 0.9% NaCl solution; then the sample was immediately frozen in liquid nitrogen and stored at −80°C until RNA extraction.

#### 2.1.2. C-Reactive Protein (C-RP) Assay

 C-RP circulating levels were determined by a high-sensitivity ELISA assay (Bender MedSystems, Vienna, Austria) on serum aliquots of all subjects included in the study. The lower detection limit was 3 pg/mL, and the overall intra-assay coefficient of variation has been calculated to be 6.9%. All samples were analyzed in duplicate.

#### 2.1.3. IL-6 Assay

The concentrations of IL-6 in plasma samples were analyzed by ELISA using a commercially available kit (Quantikine; R&D Systems, Minneapolis, MN), as described elsewhere [20]. The lower detection limit of IL-6 assay was <0.70 pg/mL, and the coefficient of variation of both inter- and intra-assay was <5%. All samples were analyzed in duplicate.

#### 2.1.4. Isolation of Adipocytes, Stromal Cells, and Preadipocytes from Adipose Tissue Fragments

Adipose tissue fragments obtained from the six noninflamed subjects and from the six inflamed patients were placed in 37°C, sterile 0.9% NaCl, 5.6 mM glucose, and 25 mM HEPES buffer with pH adjusted to 7.4 and containing 50 U penicillin/mL plus 50 mg streptomycin/mL. The adipose tissue fragments were minced under sterile conditions and digested in Krebs-Ringer bicarbonate buffer supplemented with 5.6 mM glucose, 50 U penicillin/mL, 50 mg streptomycin/mL, and 17 mg type I collagenase/10 g adipose tissue (Worthington Biochemical, Lakewood, NJ). Digestion was for 75 min at 37°C, with rotary agitation at 40 rpm. The isolated cells were filtered through a single layer of chiffon, and the isolated adipocytes were allowed to float for 5 min at 37°C. The fluid under the floating adipocytes (containing stromal fraction) was transferred to a conical polypropylene 50-mL centrifuge tube. For the adipocytes, 1 mL of cell suspension of adipose cells was suspended in each tube containing specific adipocyte medium (Zen-Bio, Research Triangle Park, NC) and incubated in the specific conditions until the cell lysis process. The stromal fraction was centrifuged at 800 g for 10 min.

For the isolation of pre-adipocytes, we first centrifuged the cell pellet at 800 × g for 10 minutes and then we washed three times with Dulbecco's modified Eagle medium : nutrient mixture F-12 (Ham) in combination 01 : 01 (DMEM: F12, Gibco-BRL, Carlsbad, California), which has been supplemented to give 15-mM NaHCO3, penicillin 50-U: mL streptomycin and 50 mg: mL. After washing, the pellet was resuspended by trituration in DMEM supplemented pre-adipocyte: F12 containing 10% fetal bovine serum (FBS, Gibco-BRL, Carlsbad, CA). The cells were then plated and incubated in a humidified incubator at 37°C in an atmosphere of 5% CO2 in air for 24 hours to allow attachment and proliferation of cells. After 24 hours, the medium was removed and replaced by specific pre-adipocyte medium (Zen-Bio, Inc., Research Triangle Park, NC) and subculture.

#### 2.1.5. Identification of Primers for Albumin and *β*-Actin

 The genome sequences corresponding to Albumin and *β*-Actin were obtained from GeneBank (http://www.ncbi.nlm.nih.gov/) to identify specific primers. We analyzed the exon sequences to establish a pair of primers (sense and antisense) able to generate amplified fragments measuring between 150 and 700 bp in length, with annealing temperature between 55 and 62°C. The sense and antisense primers were selected to include at least one intron to prevent genomic DNA contamination during amplification. The selected primer is shown in Table 1.

#### 2.1.6. RNA Isolation and Expression Analysis by Reverse Transcription-Polymerase Chain Reaction (RT-PCR)

 The isolated cells were pulverized with a blender. Total RNA was extracted by the guanidinium thiocyanate technique. Four *μ*g of total RNA were subjected to cDNA synthesis for 1 h at 37°C using the ‘‘Ready to go You-Primer First-Strand Beads” kit (Amersham Pharmacia Biotech, Piscataway, NJ, code 27-9264-01) in a reaction mixture containing 0.5 *μ*g oligo-dT (Amersham Pharmacia Biotech cod. 27-7610-01). PCR amplification of cDNA was performed in a reaction mixture containing 4 *μ*L of cDNA sample and different primer sets (20 p/mol each). The amplification of Albumin gene and human *β*-actin gene, as an internal control, was achieved using 2 primer sets. After an initial denaturation at 94°C for 5 min, PCR reactions were carried out using 30 cycles of 94°C for 1 min, the temperature for annealing for 1 min, and 72°C for 1 min in a Perkin Elmer Cetus 480 thermal cycler (Perkin Elmer). PCR products were separated by gel electrophoresis on a 2% agarose gel and stained with ethidium bromide. The bands obtained were quantified by densitometric analysis. All signals were normalized to the mRNA levels of the housekeeping gene glyceraldehyde-3-phosphate dehydrogenase and expressed as a ratio.

#### 2.1.7. Real-Time PCR

Real-time quantitative PCR analysis for albumin gene was performed on cell lysate (both adipocytes and stromal fraction) using ABI prism 7500 (Applied Biosystems, Foster City, CA) and the 5-exonuclease assay (TaqMan technology). The cDNA, synthesized as described above, was used for real-time PCR performed in 96-well optical reaction plates with cDNA equivalent of 100 ng of RNA in a volume of 25 *μ*L reaction containing 1x Taqman Universal Master Mix, optimized concentrations of FAM-labeled probe, and specific forward and reverse primer for Albumin gene from Assay on Demand (Applied Biosystems). Controls included RNA subjected to RT-PCR without reverse transcriptase and PCR with water replacing cDNA. The results were analyzed using a comparative method, and the values were normalized to the *β*-actin expression and converted into fold change based on a doubling of PCR product in each PCR cycle according to the manufacturer's guidelines, as previously described.

#### 2.1.8. Western Blot

Homogenates of adipocytes and preadipocytes were prepared in radioimmune precipitation assay buffer (1X PBS, 1% NP-40, 0.5% sodium deoxycholate, 0.1% SDS containing phenylmethylsulfonyl fluoride, aprotinin, sodium orthovanadate and protease inhibitor tablet) (completeTM Mini, Boehringer-Mannheim) containing antipain dihydrochloride (50 mg/mL), bestatin (40 mg/mL), chymostatin (60 mg/mL), E-64 (10 mg/mL), leupeptin (0.5 mg/mL), pepstatin (0.7 mg/mL), phosphoramidon (300 mg/mL), Pefabloc SC (1 mg/mL), EDTA disodium salt (0.5 mg/mL), and aprotinin (2 mg/mL)]. The cells were centrifuged at 14,000 × g for 30 min at 4°C and protein concentrations were determined with the Bradford assay [21] using bovine serum albumin as a standard. For immunoblotting, protein (100 *μ*g) was subjected to 8% SDS-polyacrylamide gel electrophoresis. Proteins were transferred to polyvinylidene difluoride (PVDF) membranes at a constant voltage of 200 V for 16 h. The PVDF membranes were blocked for 1 h in 5% nonfat dried milk in TBS-0.1% tween buffer (25 mM Tris-HCl, 0.2 mM NaCl; 0.1% Tween 20 (v/v) pH 7.6) (TBST). The membrane was washed 2X with TBST and then incubated overnight at 4°C with the respective primary antibodies. Albumin antibody was from Cell Signaling (Beverly, MA); GADPH antibodies were obtained from Santa Cruz Biotechnology. After extensive washing of membrane with TBST buffer, anti-rabbit immunoglobulin conjugated with horseradish peroxidase was added at a 1 : 5000 dilution and incubated for 1 h at room temperature. An enhanced chemiluminescence kit (Amersham, NJ) was used to identify protein expression.

### 2.2. Measurement of Albumin and Body Fat Mass in Healthy and Inflamed Patients

In sixty-three healthy (noninflamed) subjects and fifty-four microinflamed (C-RP: 5.24 ± 2.26 mg/L) ESRD patients undergoing RDT we determined circulating albumin levels (by nephelometry), Body Weight (BW) to the nearest 50 g (by using a calibrated balance beam scale), Body Mass Index (BMI), calculated as the ratio body weight/height^2^ (in kg/m^2^), and body composition, assessed by conventional bioelectrical impedance analysis (BIA) and by bioelectrical impedance vector analysis (BIVA), as previously described [22]. Resistance (*R*) and reactance (*X*_*c*_) were measured by a single-frequency 50 kHz bioelectrical impedance analyzer (BIA 101 RJL, Akern Bioresearch, Firenze, Italy) according to the standard tetrapolar technique, by applying the software provided by the manufacturer, which incorporated validated predictive equations for total body water (TBW), fat mass (FM), fat free mass (FFM), and extracellular water (ECW) [23, 24]. The same investigators performed anthropometry and BIA measurements. Soft tissue hydration of individual subjects was evaluated by BIVA. *R* and *X*_*c*_ were normalized by the height of subjects (*R*/*H* and *X*_*c*_/*H*) and the resulting vectors were plotted on a graph reporting the gender-specific 50th, 75th, and 95th tolerance ellipses of similar vectors calculated from a reference healthy population. According to the *RX*_*c*_ graph method, vectors falling within the reference gender-specific 75th tolerance ellipse indicated normal hydration; short vectors (below the lower pole of the 75th tolerance ellipse) indicated overhydration and long vectors (above the upper pole of the 75th tolerance ellipse) indicated under-hydration [25]. The length of the vector was calculated as |*Z* | = √[(*R*/*H*)2 + (*X*_*c*_/*H*)2] and the phase angle of the vector as the arctan of *X*_*c*_/*R*.

### 2.3. Statistical Analysis

Statistical analysis was performed by using unpaired *t*-test and ANOVA (followed by Bonferroni post hoc test) and linear regression analysis. Results are expressed as means ± SD; statistical significance was defined as *P* < 0.05.

## 3. Results

### 3.1. Quantization of Albumin mRNA and Protein in Adipocytes and Preadipocytes

#### 3.1.1. Patients Selection and Adipose Tissue Samples

Demographic, anthropometric, and biochemical baseline data of all enrolled subjects are reported in Table 2. No significant difference was observed in sex, age, body weight, BMI, waist circumference, and waist-to-hip ratio between the two different groups. On the contrary, plasma C-RP and IL-6 levels were much higher in patients with chronic inflammatory diseases than in healthy (noninflamed) subjects (*P* < 0.01).

#### 3.1.2. Albumin Gene Expression by RT-PCR in the Adipocyte and Preadipocyte

**Figure 1:**
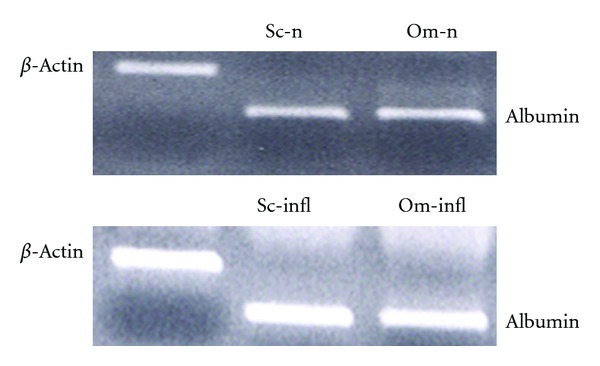
Albumin gene expression obtained by RT-PCR in subcutaneous (Sc) and Omental (Om) adipocytes from normal healthy subjects (Sc-n and Om-n, resp.) and inflamed patients (Sc-infl and Om-infl, resp.). *β*-Actin gene expression is also reported as housekeeping. The image is representative of all the experiments.

RT-PCR showed that the adipocytes extracted from all the districts of adipose tissue (omental and subcutaneous) of noninflamed controls and inflamed patients expressed the gene of albumin (Figure 1); the mRNA for this marker was found in the adipocytes and pre-adipocytes of all fragments of adipose tissue.

#### 3.1.3. Albumin Gene Expression by Real-Time PCR in the Adipocyte and Preadipocyte

**Figure 2:**
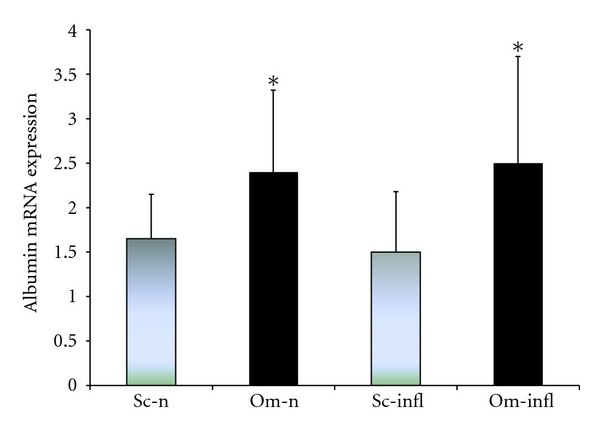
Albumin gene expression in the adipocytes from different sites of adipose tissue, obtained by real-time PCR analysis. The columns show the relative expression of albumin mRNA in subcutaneous (Sc, gray bars) and omental (Om, black bars) adipose tissue fragments of healthy noninflamed subjects (Sc-n and Om-n, resp.) compared with those obtained in both Sc and Om adipose tissue of inflamed patients (Sc-infl and Om-infl, resp.). The values on the *y*-axis represent arbitrary units derived from the mean expression value for albumin gene. Values are expressed in arbitrary units as means ± S.D. of 12 experiments. *P < 0.05 compared with Sc-infl and Sc-n.

**Figure 3:**
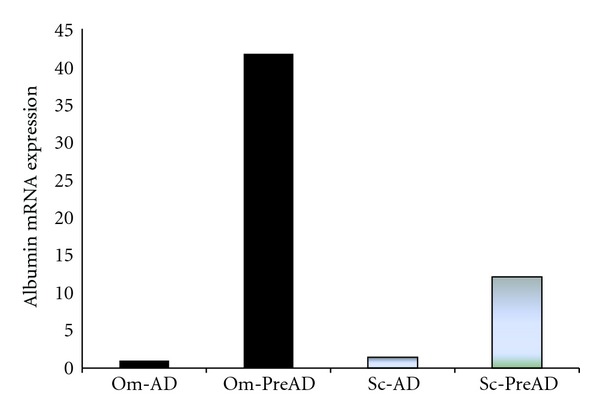
Albumin gene expression in the adipocytes and pre-adipocytes from different sites of adipose tissue of a single noninflamed subject, as representative of six experiments for all the subjects enrolled, obtained by real-time PCR analysis. The columns show the relative expression of albumin mRNA in Sc (gray bars) and Om (black bars) adipocyte (Sc-AD and Om-AD) and preadipocyte (PreAD) of a single healthy subject, as representative. The values on the *y*-axis represent arbitrary units derived from the mean expression value for albumin gene.

We did not find any statistically significant difference in albumin gene expression between inflamed and noninflamed patients in adipocytes drawn from both Sc and Om adipose tissue. As Figure 2 shows, we only observed an higher omental adipocyte albumin than subcutaneous one in each group. We concluded that inflammation does not modulate albumin gene expression in the adipocyte, but probably further studies are required to confirm this result.  Figure 3 shows the different albumin gene expression, studied by real-time PCR, in adipocytes and pre-adipocytes obtained from either Sc or Om fragments of adipose tissue drawn from noninflamed subjects. Pre-adipocyte albumin gene expression was higher then the adipocyte one in both Om and Sc fragments and this difference was statistically significant. We also observed that albumin gene expression in the pre-adipocyte extracted from fragments of Om adipose tissue was significantly higher than in preadipocyte from Sc fragments. No difference was found between the adipocytes from different districts (Figure 3).

#### 3.1.4. Albumin Protein Expression in the Adipocytes and Preadipocytes Obtained from the Different Types of Adipose Tissue Studied by Western Blot

**Figure 4:**
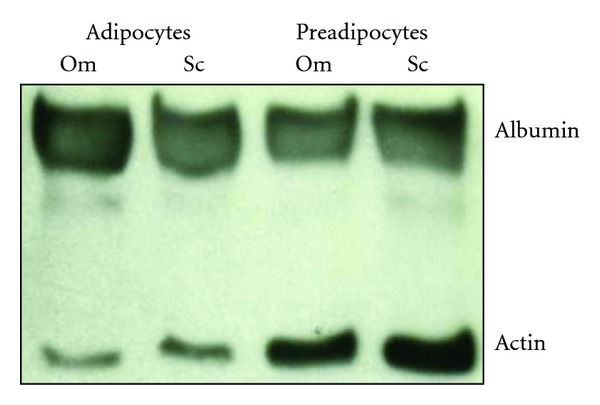
Representative immunoblot shows the albumin expression in Om and Sc adipocytes and preadipocytes isolated from a noninflamed control. Actin was used as loading control.

**Figure 5:**
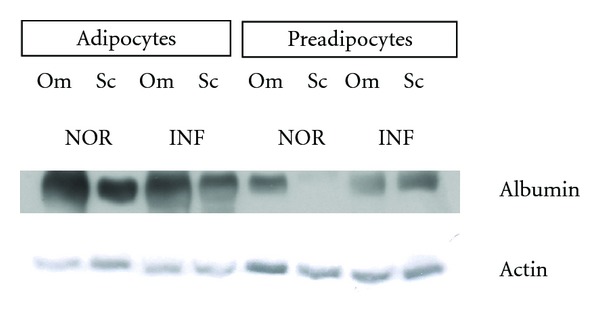
Representative immunoblot shows the albumin expression in Om and Sc adipocytes and preadipocytes isolated from both inflamed patients (INF) and noninflamed controls (NOR). Actin was used as loading control.

Finally, we investigated whether at the protein level adipocytes and pre-adipocyte were also able to express albumin in all the subjects. As shown in Figures 4 and 5, Western Blot analyses showed the presence of albumin in both adipocytes and pre-adipocytes suggesting that both adipocytes and pre-adipocytes can synthesize albumin in Sc as well as in visceral adipose tissue, both in healthy noninflamed subjects and inflamed patients.

### 3.2. Correlations between Albumin Circulating Levels and Fat Mass in Both Noninflamed Subjects and Inflamed Patients

**Figure 6:**
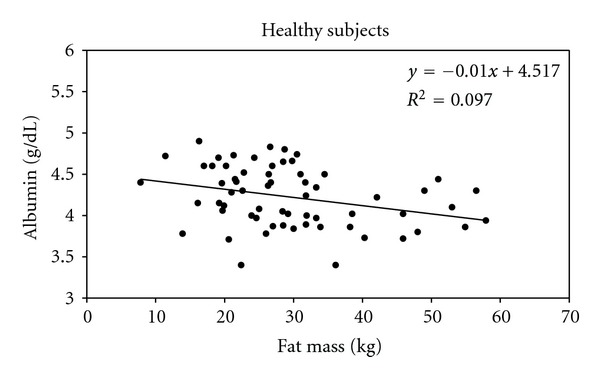
Negative relationship between Albumin circulating levels and Fat Mass. The Albumin circulating levels and Fat Mass were measured in 63 noninflamed patients. Correlation is significant at the 0.05 level (2-tailed). *R* = −0.312.

**Figure 7:**
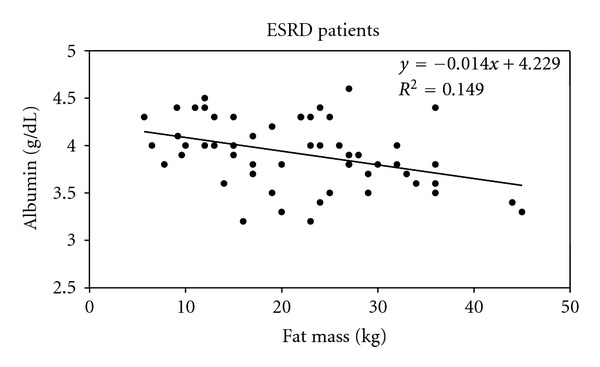
Negative relationship between Albumin circulating levels and Fat Mass. The Albumin circulating levels and Fat Mass were measured in 54 inflamed ESRD (End-Stage Renal Disease) patients. Correlation is significant at the 0.01 level (2-tailed). *R* = −0.391.

We first investigated the relationship between plasma Albumin levels and body fat mass (FM) in 63 healthy subjects who underwent BIA excluding from statistical analysis over- and hypohydrated subjects (vectors falling below the lower pole or vectors falling above the upper pole of the 75% tolerance ellipse) evaluated by BIVA method. As shown in Figure 6, a significant negative correlation was observed between plasma albumin levels and FM (*R* = −0.312, *P* < 0.05 (2-tailed)). Plasma albumin concentrations were significantly lower in those subjects with higher fat mass. Then, we studied the same relationship in 54 microinflamed ESRD patients undergoing regular dialysis therapy (RDT). As shown in Figure 7, a significant negative correlation was observed between plasma albumin levels and FM (*R* = −0.391, *P* < 0.01 (2-tailed)). Plasma albumin concentrations were significantly lower in those ESRD patients with higher fat mass.

## 4. Discussion

Albumin is the most abundant plasma protein produced by liver cells. To date, no data are present in the literature on albumin expression in mature adipocytes. In the present study, we found, for the first time, a clear albumin expression in human mature adipocytes. In addition, on the basis of our results, we can also reasonably affirm that differentiated adipocytes are probably able to synthesize albumin.

Albumin gene expression resulted significantly lower in the adipocytes than in the pre-adipocytes; in particular, it was 42 e 12 times lower in Om and Sc adipocyte, respectively, as compared with the pre-adipocyte (Figure 3). In this way, the omental pre-adipocyte represents the most active cell in albumin gene expression.

The presence of albumin in the pre-adipocyte is not a novelty. In fact, Yoo and Lee investigated the role of albumin in adipocyte differentiation, by using pre-adipocytes cell lines such as 3T3-L1 [26]. This is a developmental process by which undifferentiated precursor cells differentiate into mature adipocytes with coordinated changes in cell morphology and gene expression. They found that albumin gene expression was significantly increased at later stages of adipocyte differentiation process and its suppression significantly inhibited lipid droplet formation [26]. The author suggests that albumin could be necessary to stabilize lipid accumulation in mature adipocyte, probably through a direct interaction with fatty acids. However, it is important to underline that these experiments were performed on murine cell lines and their results cannot correspond to human cell data.

In a previous paper, we studied the involvement of adipose cells in patients with chronic inflammatory disease. We found that not only C-RP gene expression was activated in adipocyte cells, but both IL-6 and IL-6 receptors gene expressions were found to be higher in inflamed patients than in controls, either in subcutaneous or intra-abdominal adipose tissue [5].

At this regard, in the present study we hypothesized that adipocytes, similarly to hepatocytes, show a different albumin gene expression in inflamed and noninflamed patients, with a lower gene expression in inflamed ones. However, our results showed no significant difference in albumin gene expression between inflamed and noninflamed patients when analyzed by Real-Time PCR (Figure 2). On the other hand, Figure 2 shows also that albumin gene expression was significantly higher in intra-abdominal than in subcutaneous adipocytes (Figure 2).

Albumin presence, as protein, together with gene expression in adipocytes raises the hypothesis that adipose tissue contribute to circulating albumin levels, as well as it happens for IL-6 [3]. To verify this hypothesis we evaluated the relationship between serum albumin and fat mass, supposing that higher fat mass corresponds to higher circulating albumin levels. However, our results did not confirm this hypothesis, but we found a negative significant correlation between albumin and fat mass both in healthy noninflamed subjects and inflamed ESRD patients, in contrast with what we expected (Figures 6 and 7). In other words, the higher the fat mass the lower was serum albumin concentration was. We suppose that higher fat mass leads to higher production of different proinflammatory cytokines, mainly IL-6, that can downregulate albumin, production in hepatocyte via endocrine way, independently of systemic inflammation. We also suppose that IL-6 produced by adipocyte could down-regulate, via an autocrine and/or paracrine signaling, albumin gene expression and production in the adipocyte itself. This mechanism could explain both the lack of modulation of albumin gene expression by systemic inflammation in the adipocyte and the negative correlation between fat mass and albumin levels either in noninflamed subjects or in inflamed ESRD patients. On the contrary, this mechanism could not operate with inflammatory proteins, such as C-RP [5].

Despite the lack of albumin gene modulation by inflammation, in this study something really new was observed: the human adipocyte, once considered a simply depot cell, is now seen as a new and active cell, that in parallel with hepatocyte, is able to produce different proteins, such as C-RP and, as novelty, albumin.

Why adipocyte shows hepatocyte-like activity is still unknown. The more fascinating hypothesis states the existence of a continuum in adipose tissue cell population that goes from multipotent stem cell to more mature progenitor pools [6], passing through the pre-adipocyte. This hypothesis might explain why adipocyte and hepatocyte share the expression of some genes, such as albumin gene. As above mentioned, adipose-tissue-derived MSC (ADMSC) displayed the capacity to differentiate into numerous cell types (muscular, neuronal, bone, adipose cells) and, interestingly, into hepatocyte-like cells [13–15]. A study showed that ADMSC could be differentiated into functional hepatocyte-like cells by the treatment of cytokine mixtures *in vitro* [16], so to become a potential source to hepatocyte regeneration or liver cell transplantation [27]. In another work, the authors showed that the undifferentiated naïve ADMSC were also positive for albumin, G-6-P, and *α*-1-antitrypsin (AAT), which are all known to be predominantly expressed in adult liver cells [28].

However, we do suppose that other, more fascinating mechanisms, apart from sharing the same origin, may explain why, to date, adipocyte and hepatocyte produce a so important protein in our organism, like albumin.

In conclusion, this preliminary study highlights for the first time a new adipocyte activity, among the others already known; however further investigations are needed to confirm and explain our results. 

## Figures and Tables

**Table 1:** 

Primers	Sense	Antisense
ALBUMIN	CTTGAATGTGCTGATGACAGG	GCAAGTCAGCAGGCATCTCAT
*β*-ACTIN	CACCATGGATGATGATATCG	TGGATAGCAACGTACATGG

Oligonucleotide sequences designed for this study.

**Table 2:** Demographic and anthropometric characteristics of the 12 subjects who underwent the adipose tissue biopsy.

	Noninflamed subjects	Chronic inflamed patients	*P* value
*N*	6	6	NS
Sex (M/F)	4/2	3/3	NS
Age, yr (range)	43.2 ± 4.0 (39–48)	42.9 ± 3.9 (34–49)	NS
Body weight, kg (range)	74.8 ± 10.6 (62.9–88.2)	75.3 ± 11.1 (63.2–90.7)	NS
BMI, kg/m² (range)	25.8 ± 1.6 (24.8–28.1)	26.3 ± 1.2 (24.6–28.7)	NS
Waist circumf., cm (range)	90.9 ± 4.6 (83–106)	92.1 ± 6.1 (79–108)	NS
Waist-to-hip ratio (range)	0.82 ± 0.11 (0.53–1.21)	0.88 ± 0.15 (0.65 ± 1.28)	NS
C-RP, mg/L (range)	2.18 ± 0.54 (1.6–3.0)	7.26 ± 3.26 (4.1–14.6)	*P* < 0.01
IL-6, pg/mL (range)	2.79 ± 1.26 (1.3–4.9)	25.19 ± 11.85 (10.3–40.3)	*P* < 0.01

Values are expressed as means ±  SD; M, male; F, female; NS, not significant; C-RP, C-reactive protein.
